# Erysipelas

**Published:** 1893-02

**Authors:** J. H. Allen

**Affiliations:** Logansport, Ind.


### ERYSIPELAS.

J. H. Allen, M. D., Logansport, Ind.

Erysipelas. This disease, though sometimes classified as a surgical disease, belongs to dermatology.

The exciting cause may be most anything. It may follow colds, chilling of the body, badly treated or neglected wounds, running sores, suppressed discharges, leucorrhœa, gonorrhœa. Suppressed rheumatism is a potent cause, catarrhal conditions badly treated, especially those treated locally.

The predisposing causes, as far as my experience goes, are three: psora, sycosis, syphilis. Psora, combined with some suppressed disease, itch, etc., are common causes.

It is found more frequently in females than in males, and usually in adult life; though I have seen a number of cases in infants occur the fourth day after birth, due to a slight abrasion of the scalp.

The simple forms usually succumb easily to homœopathic treatment, but when it is treated topically, bad, if not disastrous results are apt to follow. Often here is the beginning of a long series of ailments. Rheumatism I will place first in the list, stubborn and persistent neuralgia, headache, amenorrhœa, ovarialgia, dyspepsia, heart, liver, or lung troubles.

I remember treating a case of neuralgia of the left hip in a young lady who had suffered with it for ten years. She complained of its being worse at menstrual period or change of weather. After prescribing for six months, with only temporary relief, I gave a dose of Psorinum$^{cm}$. In six hours a large erysipelatous spot made its appearance over the seat of pain. It was of the vesicular form. No medicine was given for four days. It spread so rapidly that I was compelled to give a dose of Rhus, as quite severe constitutional symptoms had developed. But one dose of the Rhus was given with complete cure of the eruption and no return of the pain, though two years have elapsed since then.

Cellulo-cutaneous or phlegmonous forms are always to be dreaded, the mortality is very great, equal, I think to puerperal petitonitis or diphtheria. There is some similarity in the heat line, starting at the normal, the temperature drops to 96 often, and almost as abruptly it rises to 104 2-5. Every tissue may be affected by this virulent poison. In the soft tissues the inflammation is often of so specific a nature that in twenty-four to thirty hours the point of attack becomes gangrenous and sloughs away, frequently causing severe hemorrhages.

In two cases I was called to treat, both were unconscious within twenty-four hours. Great physical and mental restlessness accompanied each case; the pains are unbearable, and of a burning character; the tissues assume a dark red appearance, blue, even black, according to the intensity of the inflammation. The œdema is usually great, infiltration rapid, pus cavities soon form, which discharge enormous quantities of pus, usually acrid, bloody, and foul smelling. They bleed at the slightest touch, dipping down into the bony structure, producing necrosis; often false granulations form, which retard a cure.

The prognosis, says the allopath school, is always grave, and generally fatal, Quinine and topical application being their stock in trade. Among the latest of local measures is clay poultices.

But my own school of medicine has something more encouraging to offer, most cases being promptly cured by a careful selection of the homœopathic remedy. There must be no delay in getting it as early as possible, as the destruction of the tissues is very rapid, and you may be able to save your patient, not only from weeks of suffering and pain, but, probably, a future operation.

I offer here a case to the profession that may be of interest in several ways; first, it was cured by the single remedy, and that in a very high potency; secondly, no application was used except cloths wrung from warm water, which excluded the air and kept the part warm; in the third place, no operation was performed for the removal of necrosed bone; and, last of all, there was no deformity.

Mr. C. S., age fifty-four, thin, dark complexion, medium height, weight one hundred and forty, nervo-bilious temperament. Previous to my being called, had suffered three weeks with a felon on the middle third of the left index finger. His sufferings during that time must have been very great, judging from the terribly swollen and discolored condition of the affected part. He was then unconscious, and had been most of the day. The delirium at times was furious, and there was much restlessness and tossing about, great fear that some one was going to kill him or that he was going to die, very thirsty in the after part of the night, prostration very great during his rational hours. In the early morning, he was so weak that he could not raise his head from the pillow. The temperature 104 and pulse 140. Arsenicum$^{cm}$ was given every half-hour until five doses were taken; then no medicine. This prescription was made at 4 P. M. Rhus was left to be given if no better in any way in six or eight hours. I called in the morning again, and found him worse. Neither medicine had had the desired effect. He had passed a terrible night, no rest for a moment. The tongue was dry and crisp, and the symptoms worse in every way. The temperature was now 105¼, the soft tissues of the whole anterior surface of the finger had sloughed away, a large blister involving most of the thumb and palmar surface of the hand was filled with serum.

Posterior portion of the hand looked like a large fungus, ready to slough away at any time, bleeding profusely on being touched. A terribly fetid odor prevailed in the room. The inflammation had extended up the arm to the shoulder, and matters were assuming a serious aspect. The pulse was becoming unsteady; the body was covered with perspiration, which was cold for a few hours, then warm again; knees were quite cold, and had been for three weeks, so the nurse told me; had to have extra covering on them. The least food produced great distress in the stomach, which was very much bloated. He constantly belched, which afforded some relief. The latter symptoms I failed to get when I made my first prescription.

I then prescribed Carbo-veg.$^{cm}$, three powders, one every hour. After taking the first dose, he fell into a refreshing sleep and slept three hours, when the temperature dropped and the malignant condition gradually left. His mental condition, previous to my giving the Carbo-veg., was terrible, and the family begged me to give him a hypodermic of Morphine, that he might get a moment's rest. Had such suggestions been followed, the results would, no doubt, have been fatal.

For sixty days I followed up this case, giving only an occasional dose of Carbo-veg. as relapses would occur. During this time, a number of pieces of necrosed bone came away without any surgical interference.

The patient made a good recovery, with no injury of the finger save a slight stiffness, which soon disappeared.

### Discussion.

Dr. Long—I take exception to the suggestion of hot-water cloths applied at intervals.

Dr. Campbell—I indorse that exception.

Dr. Pease—I also indorse the exception.

Dr. J. H. Allen—I did not have to use the hot cloths; I found the right remedy.

